# Glutamine Oxidation in Mouse Dorsal Root Ganglia Regulates Pain Resolution and Chronification

**DOI:** 10.1523/JNEUROSCI.1442-24.2024

**Published:** 2024-10-08

**Authors:** Md Mamunul Haque, Panjamurthy Kuppusamy, Ohannes K. Melemedjian

**Affiliations:** ^1^Department of Neural and Pain Sciences, University of Maryland School of Dentistry, Baltimore, Maryland 21201; ^2^UM Center to Advance Chronic Pain Research, Baltimore, Maryland 21201; ^3^UM Marlene and Stewart Greenebaum Comprehensive Cancer Center, Baltimore, Maryland 21201

**Keywords:** ASCT2, chronic pain, dorsal root ganglia, hyperalgesic priming, metabolism, nerve growth factor

## Abstract

Chronic pain remains a significant health challenge with limited effective treatments. This study investigates the metabolic changes underlying pain progression and resolution, uncovering a novel compensatory mechanism in sensory neurons. Using the hyperalgesic priming model in male mice, we demonstrate that nerve growth factor (NGF) initially disrupted mitochondrial pyruvate oxidation, leading to acute allodynia. Surprisingly, this metabolic disruption persisted even after the apparent resolution of allodynia. We discovered that during the resolution phase, sensory neurons exhibit increased glutamine oxidation and upregulation of the major glutamine transporter ASCT2 in dorsal root ganglia. This compensatory response plays a crucial role in pain resolution, as demonstrated by our experiments. Knockdown of ASCT2 prevents the resolution of NGF-induced allodynia and precipitates the transition to a chronic state. Furthermore, we show that the glutamine catabolite α-ketoglutarate attenuated glycolytic flux and alleviated allodynia in both acute and chronic phases of the hyperalgesic priming model. The importance of ASCT2 is further confirmed in a translational model, where its knockdown prevented the resolution of allodynia following plantar incision. These findings highlight the pivotal role of metabolic changes in pain resolution and identify ASCT2-mediated glutamine metabolism as a potential therapeutic target for chronic pain. Understanding these endogenous mechanisms that promote pain resolution can guide the development of novel interventions to prevent the transition pain from acute to chronic.

## Significance Statement

Chronic pain is a widespread health issue with limited effective treatments. This study unveils a critical metabolic mechanism in sensory neurons that determines whether acute pain resolves or becomes chronic. We discovered that pain resolution depends on a compensatory increase in glutamine metabolism, mediated by the transporter ASCT2, rather than normalization of initial metabolic disruptions. This finding significantly advances our understanding of pain chronification and identifies a novel therapeutic target. By elucidating how the body naturally resolves pain, we open new avenues for developing treatments that could prevent acute pain from transitioning to chronic pain or treat existing chronic pain. This research has the potential to transform pain management strategies and improve quality of life for millions of pain sufferers.

## Introduction

Chronic pain is a debilitating condition that affects millions of people worldwide. Despite significant advances in our understanding of pain mechanisms, effective treatments for chronic pain remain limited. Recent evidence suggests that alterations in cellular metabolism, particularly in sensory neurons, may play a crucial role in the development and maintenance of chronic pain states (Ludman and Melemedjian, 2019a,b; Haque et al., 2024; Kuppusamy et al., 2024). Understanding the mechanisms of pain resolution can help develop novel therapies for treating chronic pain and preventing the transition from acute to chronic pain states.

Nerve growth factor (NGF) has been demonstrated to reprogram sensory neuron metabolism to suppress mitochondrial pyruvate oxidation and enhance lactate extrusion, thereby extending nociceptive sensitization (Haque et al., 2024). This metabolic reprogramming was driven by increased expression of lactate dehydrogenase A (LDHA) and pyruvate dehydrogenase kinase 1 (PDHK1) at 24 h post-NGF treatment. Pharmacological inhibition or knockdown of these enzymes reversed the NGF-induced metabolic changes and accelerated the resolution of allodynia. The study also revealed that NGF appears to engage distinct pain phases: an initial acute phase likely mediated by posttranslational modifications, a second delayed phase involving metabolic reprogramming, and a final resolution phase where pain sensitivity returns to the baseline. Crucially, the study demonstrated that disruption of mitochondrial pyruvate oxidation was sufficient to cause allodynia lasting months, unlike NGF-induced allodynia which resolved within 72 h (Haque et al., 2024).

Despite these findings, it remains unclear whether the resolution of NGF-induced allodynia is due to the normalization of mitochondrial pyruvate oxidation or the engagement of a compensatory mechanism that restores sensory neuron function despite persistent metabolic dysfunction. In this study, we utilize the hyperalgesic priming model to investigate the underlying mechanisms responsible for the resolution of NGF-induced allodynia and the potential role of compensatory metabolic adaptations in this process. The hyperalgesic priming model is well characterized and used to study the neuroplasticity underlying persistent nociceptor sensitization and the transition of acute pain to chronic (Reichling and Levine, 2009; Melemedjian et al., 2010, 2014; Melemedjian and Khoutorsky, 2015; Ferrari et al., 2015a). The model is comprised of three phases: (1) the acute or induction phase, which causes transient tactile hypersensitivity in response to various pronociceptive compounds and resolves within a week; (2) the primed phase, where the animals are not hypersensitive, that can last for at least 3 weeks; and (3) the chronic or maintenance phase, which can be expressed by plantar injection of PGE_2_ or other stimuli and has been demonstrated to last for at least 2 months (Aley et al., 2000; Melemedjian et al., 2010, 2014; Ferrari et al., 2015a,b). We chose this model since it lacks injury to major peripheral nerves and typically resolves within a week, making it suitable for studying the neuroplasticity underlying persistent pain sensitization and the transition of acute pain to chronic.

We tested the hypothesis that the resolution of NGF-mediated allodynia may occur independently of the recovery of pyruvate oxidation, suggesting the existence of alternative metabolic pathways that can compensate for the deficits in mitochondrial function. To do this, we examine the temporal dynamics of mitochondrial pyruvate oxidation in dorsal root ganglion (DRG) neurons during the different phases of nociceptive sensitization. We found that mitochondrial pyruvate oxidation does not normalize even after allodynia returns to baseline levels. Instead, we uncover a novel compensatory mechanism involving enhanced glutamine oxidation and the upregulation of the major glutamine transporter ASCT2/SLC1A5 (Solute Carrier Family 1, Member 5). We demonstrate that this adaptive metabolic response is critical for the resolution of NGF-mediated allodynia and that its disruption can precipitate the transition to a chronic hypersensitivity state. Disruption of ASCT2 expression also prevented the resolution of postincisional pain, validating these findings in a translational model.

## Materials and Methods

### Experimental animals

Pathogen-free, adult male Institute for Cancer Research (ICR) mice (3–4 weeks old; Envigo) were housed in temperature- (23 ± 3°C) and light (12 h light/12 h dark cycle; lights on 07:00–19:00)-controlled rooms with standard rodent chow and water available *ad libitum*. Animals were randomly assigned to treatment or control groups for the behavioral experiments. Animals were initially housed five per cage. All behavioral experiments were performed by experimenters who were blinded to the experimental groups and treatments. The Institutional Animal Care and Use Committee of the University of Maryland approved all experiments. All procedures were conducted in accordance with the Guide for Care and Use of Laboratory Animals published by the National Institutes of Health (NIH) and the ethical guidelines of the International Association for the Study of Pain.

### Mechanical testing

Male ICR mice were placed in acrylic boxes with wire mesh floors, and baseline mechanical withdrawal thresholds of the left hindpaw were measured after habituation for 1 h using the up–down method (Chaplan et al., 1994). After determining the baseline withdrawal thresholds of mice hindpaw using von Frey filaments, the mice received intraplantar (IPL) vehicle or NGF (0.1 ng in 25 µl PBS, Millipore Sigma, catalog #01-125). For experiments with intraperitoneal (IP) injections, oxamate (500 mg/kg, Millipore Sigma, catalog #O2751), dimethyl-alpha-ketoglutarate (DKG; 300 mg/kg, Millipore Sigma, catalog #-34963), or dichloroacetate (DCA; 100 mg/kg, Millipore Sigma, catalog #347795) was injected at the indicated time points. For experiments with intrathecal (IT) siRNA treatments, at the indicated time points, mice were tested before IT injection, and the injections were done between the L4 and L5 vertebrae under isoflurane anesthesia. Negative control (Millipore Sigma, catalog #SIC001), LDHA (Millipore Sigma, SASI_Mm01_00049543), ASCT2 (Millipore Sigma, SASI_Mm01_00186887), and PDHK1 (Millipore Sigma, SASI_Mm01_00042115) HPLC-purified siRNAs were injected at a dose of 1 µg in 5 l of i-Fect (Neuromics, catalog #NI35150). HPLC-purified pyruvate dehydrogenase phosphatase 1 (PDP1) siRNA (1 µg in 5 µl of i-Fect, Millipore Sigma, SASI_Mm01_00038882) or control siRNA was injected following testing the baseline thresholds.

### Conditioned place aversion (CPA)

The CPA apparatus consists of three opaque chambers separated by manual doors. A center chamber (60 mm W × 60 mm D × 150 mm H) connects the two end chambers that are identical in size (150 mm W × 150 mm D × 150 mm H) but can be distinguished by the texture of the floor (circular opening vs diamond pattern mesh), wall color (black walls white ceiling vs white walls black ceiling), and olfactory cues (vanilla vs cherry ChapStick applied on a cotton swab). Movement of mice and time spent in each chamber were monitored and recorded using custom-built infrared sensors and software. Preconditioning was performed 48 h postplantar PGE_2_ treatment for 30 min when mice were exposed to the environment with full access to all chambers. A single-trial conditioning protocol was used in the experiments. On conditioning day (Day 2), mice first received vehicle control (intraperitoneal saline) paired with a randomly chosen chamber in the morning and, 3–4 h later, intraperitoneal glucose (2 g/kg in saline) (Andrikopoulos et al., 2008) paired with the other chamber. Food was withheld for 3–4 h prior to the glucose administration. The normal plasma concentration of glucose in mice in ∼7 mM where fasting for 3–4 h does not significantly alter these levels. After glucose injection (2 g/kg), the plasma level rapidly reaches to ∼20 mM for ∼30 min, and within 60 min, the glucose levels return back to normal (Andrikopoulos et al., 2008). During the conditioning, mice were allowed to stay only in the paired chamber without access to other chambers for 30 min immediately following saline or glucose injection. On the test day, 20 h after the glucose pairing, mice were placed in the middle chamber of the CPA box with all doors open, so animals can have *ad libitum* access to all chambers. Movement and duration of each mouse spent in each chamber were recorded for 30 min for analysis of chamber aversion. Mice received vehicle or oxamate (500 mg/kg, i.p.) 2 h prior to the glucose administration. DKG (300 mg/kg, i.p.), DCA (100 mg/kg, i.p.), or vehicle was administered 1 h prior to glucose administration.

### Plantar incision and behavioral testing

Before surgery, all animals were assessed for paw withdrawal thresholds. A mouse model of incisional pain was used for this study (Banik et al., 2006). A 5 mm longitudinal incision was made with a Number 11 blade through the skin, fascia, and muscle of the plantar aspect of the hindpaw in isoflurane-anesthetized mice. The skin was apposed with one suture of 5 mm silk. Sham controls underwent the same procedure but without the incision. Animals were allowed to recover for 24 h, and then paw withdrawal thresholds were measured.

### DRG dissociation

L4–L6 DRGs were excised aseptically at the indicated time points and placed in Hank's buffered salt solution (Thermo Fisher Scientific, catalog #14170112) on ice. The ganglia were dissociated enzymatically with collagenase A (1 mg/ml, 25 min, Millipore Sigma, catalog #10103578001) and collagenase D (1 mg/ml, Millipore Sigma, catalog #11088858001) with papain (30 U/ml, Millipore Sigma, catalog #10108014001) for 20 min at 37°C. To eliminate debris and large-diameter sensory neurons, we used 70 μm (Thermo Fisher Scientific, catalog #087712) cell strainers. The dissociated cells were resuspended in DMEM/F12 (Thermo Fisher Scientific, catalog #10565042) containing 1× pen–strep (Thermo Fisher Scientific, catalog #15070063) and 10% fetal bovine serum (Millipore Sigma, catalog #F2442). The cells were plated in Seahorse XFp Cell Culture Miniplate (Agilent Technologies, catalog #103025-100), BioCoat poly-D-lysine-coated six–well plates (Corning, catalog #356413), or poly-D-lysine-coated, glass-bottom 35 mm dishes (Mattek Life Sciences, catalog #P35GC-1.5-10). The primary afferent cultures were incubated overnight at 37°C in a humidified 95% air/5% CO_2_ incubator.

### Metabolic assays

The metabolic changes were characterized by analyzing the glycolysis and oxidative phosphorylation rates of sensory neurons using the extracellular flux analyzer, Seahorse XFp (Agilent Technologies).

#### Mito Stress Test

L4–L6 DRGs were dissected, acutely dissociated, and incubated in the XF analyzer plates overnight which allows for the neurons to adhere to the bottom of the plates. The Mito Stress Test was performed in DMEM medium (Millipore Sigma, catalog #D5030) that contained glucose (10 mM), glutamine (2 mM), and pyruvate (1 mM). During the Mito Stress Test, baseline oxygen consumption rate (OCR) measurements were followed by the addition of compounds that target components of the electron transport chain in the mitochondria to reveal key parameters of oxidative phosphorylation. The compounds oligomycin (5 µM, Millipore Sigma, catalog #75351), carbonyl cyanide-p-trifluoromethoxyphenylhydrazone (FCCP; 4 µM, Millipore Sigma, catalog #C2920), and a mix of rotenone (2 µM, Millipore Sigma, catalog #R8875) and antimycin A (2 µM, Millipore Sigma, catalog #A8674) are serially injected to measure ATP-linked respiration, maximal respiration, and nonmitochondrial respiration, respectively. Proton leak and spare respiratory capacity are then calculated using these parameters (Divakaruni et al., 2014; Yang et al., 2014).

#### Glycolysis stress test

The dissociated L4–L6 DRG neurons were incubated in DMEM medium (Millipore Sigma, catalog #D5030) without glucose or pyruvate and the baseline extracellular acidification rate (ECAR) was measured. The cells were deprived of glucose for ∼30–40 min. It should be noted that the DMEM medium contains amino acids that the cells utilize to maintain energetics. In addition to amino acids, the medium contains phosphates where both can serve as mild pH buffers. A saturating concentration of glucose (10 mM, Millipore Sigma, catalog #G8769) is injected to measure the glycolysis rate which is followed by injection of oligomycin (5 µM) which inhibits mitochondrial ATP production and shifts the energy production to glycolysis, with the subsequent increase in ECAR revealing the cellular maximum glycolytic capacity. The final injection is 2-deoxyglucose (50 mM, Millipore Sigma, catalog #D8375), which inhibits glycolysis by competitively binding to hexokinase, the first enzyme in the glycolytic pathway. The resulting decrease in ECAR confirms that the ECAR produced in the experiment is due to glycolysis. The difference between glycolytic capacity and glycolysis rate defines glycolytic reserve. ECAR, prior to glucose injection, is referred to as nonglycolytic acidification, caused by processes in the cell other than glycolysis (Divakaruni et al., 2014; Yang et al., 2014).

#### Pyruvate oxidation assay

Dissociated L4–L6 primary afferents were incubated in DMEM (Millipore Sigma, catalog #D5030) without glutamine, and baseline OCR measurements were followed by the addition of pyruvate (1 mM, Millipore Sigma, catalog #S8636). The neurons were then treated with the PDHK inhibitor, DCA (20 mM). At the end of the assay, the mitochondrial pyruvate transporter inhibitor, UK5099 (10 µM, Millipore Sigma, catalog #PZ0160), was added. The OCR values obtained following the addition of UK5099 were subtracted from each of the other values to determine the pyruvate oxidation-dependent OCR.

#### Normalization of metabolic assays

After the completion of the metabolic assays, protein content was determined using Pierce BCA Protein Assay Kit (Thermo Fisher Scientific, catalog #23225). The data obtained from the metabolic assays were normalized to the protein content.

### Calcium imaging

Dissociated DRG cells were loaded with Fluo4-AM (1 µM, Thermo Fisher Scientific, catalog #F14217) for 20 min at 37°C in DMEM (Millipore Sigma, catalog #D5030) with glucose (10 mM). The cells were then transferred to a recording chamber placed on an inverted microscope (Olympus IX73). Images were captured using Micro-Manager 1.4 and analyzed with Fiji ImageJ 1.52c software (NIH). Neurons measuring between 20 and 35 µm in diameter were analyzed. The area under the curve (AUC) was determined using GraphPad Prism 10. Cells were pretreated with vehicle or DKG (1 mM) prior to the addition of UK5099 (5 µM) at 100 s time point.

### Western blotting

Protein was extracted from the L4–L6 DRGs in lysis buffer (50 mM Tris–HCl, 1% Triton X-100, 150 mM NaCl, and 1 mM EDTA) at pH 7.4 containing protease and phosphatase inhibitor mixtures with an ultrasonicator on ice and cleared of cellular debris by centrifugation at 14,000 relative centrifugal force for 15 min at 4°C. Fifteen micrograms of protein per well were loaded and separated by standard 7.5 or 10% SDS-PAGE. Proteins were transferred to Immobilon-P membranes (Millipore Sigma, catalog #IPVH00010) and then blocked with 5% dry milk for 3 h at room temperature. The blots were incubated with primary antibody overnight at 4°C and detected the following day with donkey anti-rabbit or goat anti-mouse antibody conjugated to horseradish peroxidase (1:10,000, Jackson ImmunoResearch Laboratories, catalog #711-036-152, catalog #115-036-062). The signal was detected by enhanced chemiluminescence on films. For assessment of phosphoproteins, membranes were stripped and reprobed for total-protein of interest for normalization. Densitometric analyses were done using the UN-SCAN-IT 7.1 software (Silk Scientific). Primary antibodies include phospho-PDH Ser300, phospho-PDH Ser293, PDP1 (1:1,000, Millipore Sigma, catalog #ABS194, ABS204, 07-1223), PDH, LDHA, PDHK1, ASCT2 (1:1,000, Cell Signaling Technology, catalog #3205, 3582, 3820, 5345), and beta-III-tubulin (1:50,000, Promega, catalog #G7121).

### Statistical analysis and data presentation

Data are based on the means and the standard error of the means (±SEM). Graph plotting and statistical analysis used GraphPad Prism Version 10 (GraphPad Software). When analyzing metabolic assays or evoked pain behavior data, two-way repeated–measure (RM) ANOVA followed by post hoc pairwise comparisons with Bonferroni’s correction was used. Calcium imaging and Western blot data were analyzed by one-way ANOVA or unpaired *t* test. The a priori level of significance at a 95% confidence level was considered at *p* < 0.05.

## Results

### NGF-induced hyperalgesic priming reveals persistent alterations in mitochondrial pyruvate oxidation despite apparent recovery of tactile thresholds

**Figure 1.**
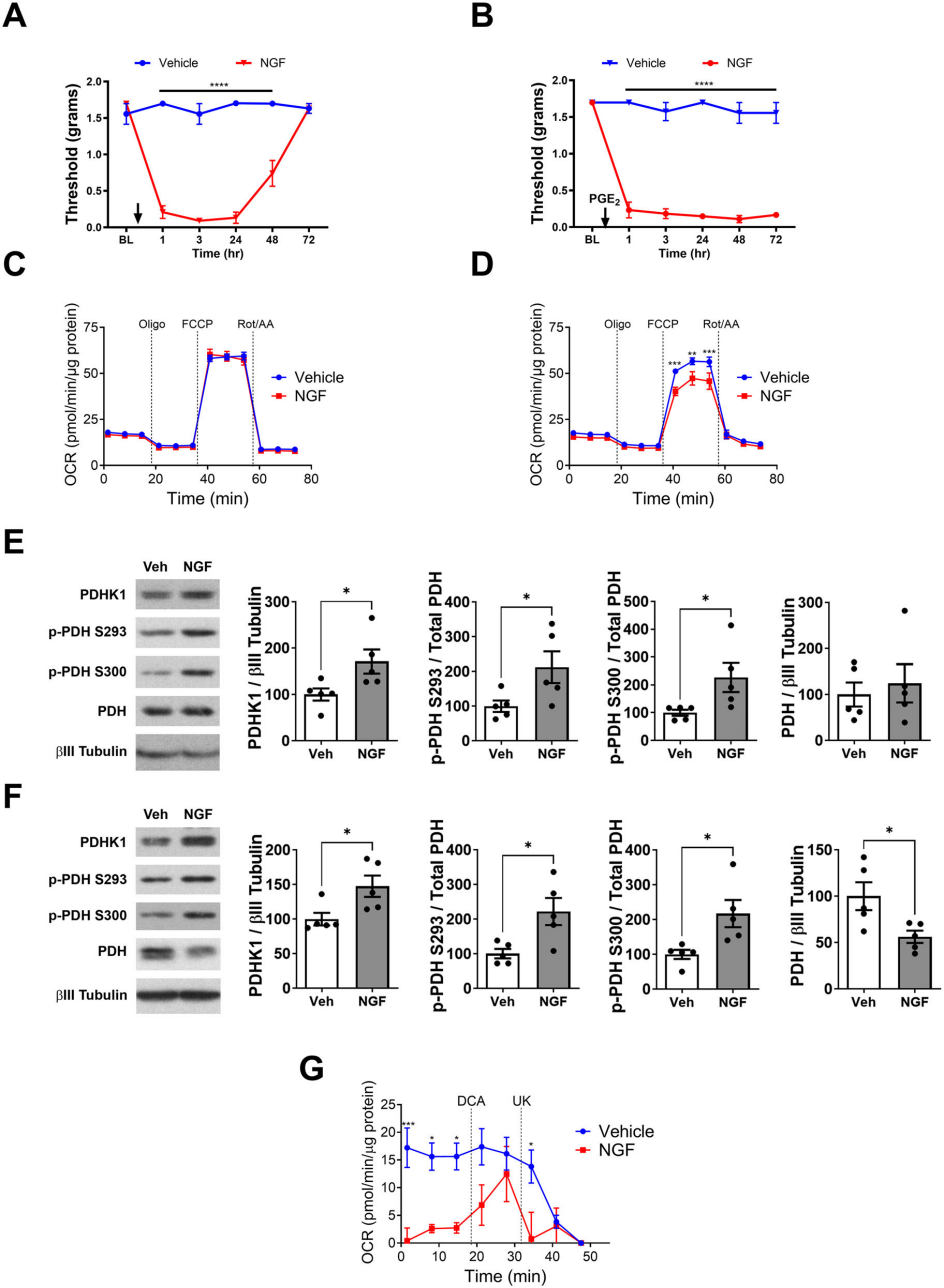
NGF-induced hyperalgesic priming model and its impact on mitochondrial function in lumbar DRGs. ***A***, Tactile thresholds measured by von Frey filaments in mice injected with IPL vehicle or NGF. NGF induces acute tactile allodynia (acute phase; vehicle vs NGF; ****p < 0.0001) that resolves within 72 h. ***B***, On Day 7, mice primed with NGF do not exhibit tactile allodynia (primed phase) but develop tactile allodynia after IPL PGE_2_ injection (chronic phase). Vehicle-treated mice do not develop allodynia post-PGE_2_ (vehicle vs NGF; ****p < 0.0001). ***C***, ***D***, Mitochondrial stress test measured by Seahorse analyzer in lumbar DRGs from NGF- or vehicle-treated mice dissected on Day 7 (***C***) or 3 d post-PGE_2_ injection (***D***). NGF injection alters mitochondrial respiration, reducing maximal respiration and spared respiratory capacity in chronic (post-PGE_2_) but not in primed state (**p < 0.01; ***p < 0.001). ***E***, ***F***, Western blot analysis of PDHK1, phospho-PDH S293, phospho-PDH S300, and total PDH in lumbar DRGs from NGF- or vehicle-treated mice on Day 7 (***E***) or 3 d post-PGE_2_ injection (***F***). NGF increases PDHK1 expression and PDH phosphorylation in both primed and chronic states and reduces PDH expression in chronic state (*p < 0.05). ***G***, Pyruvate oxidation in lumbar DRGs from NGF- or vehicle-treated mice on Day 7. NGF priming reduces basal pyruvate oxidation, and this effect is reversed by the PDHK inhibitor DCA (20 mM). The mitochondrial pyruvate carrier inhibitor UK5099 (UK, 10 µM) was added at the end of the assay to measure pyruvate-dependent OCR (*p < 0.05; ***p < 0.001).

In this study, we employed the hyperalgesic priming model to investigate the impact of NGF on nociception and mitochondrial function in lumbar DRGs. IPL injection of NGF (0.1 ng) induced significant allodynia compared with the vehicle-treated group, which resolved within 72 h (Fig. 1*A*), representing the acute phase of the model. Two-way RM ANOVA revealed a main effect for the time (*F*_(5, 40)_ = 30.86; *p *< 0.0001) and group (*F*_(1, 8)_ = 424.9; *p* < 0.0001). Post hoc pairwise comparisons with Bonferroni’s correction revealed a significant (****p < 0.0001) difference in allodynia between the mice treated with NGF and vehicle (five mice/group). On Day 7 post-NGF injection, during the primed phase, tactile thresholds returned to baseline levels, comparable with naive mice. However, when both vehicle- and NGF-pretreated mice were challenged with IPL PGE_2_, the NGF-primed mice developed profound allodynia (Fig. 1*B*), precipitating the chronic phase, which is known to persist for months. Two-way RM ANOVA revealed a main effect for the time (*F*_(5, 40)_ = 35.30; *p* < 0.0001) and group (*F*_(1, 8)_ = 611.1; *p* < 0.0001). Post hoc pairwise comparisons with Bonferroni’s correction revealed a significant (****p < 0.0001) difference in allodynia between the mice treated with NGF and vehicle (five mice/group).

Previous studies have shown that NGF disrupts mitochondrial pyruvate oxidation during the acute phase, leading to a reduction in the OCR in response to FCCP (Haque et al., 2024). Interestingly, mitochondrial stress tests performed on DRGs dissected on Day 7 postvehicle or NGF treatment revealed that the NGF-treated group was indistinguishable from the vehicle-treated group (Fig. 1*C*), suggesting a recovery in mitochondrial function as the allodynia resolved. Moreover, DRGs dissected 72 h post-PGE_2_ treatment (chronic phase) demonstrated a reduction in FCCP-induced OCR in the NGF-primed mice (Fig. 1*D*), indicating that the disruption of mitochondrial oxidation may contribute to the chronic phase. Two-way RM ANOVA revealed a main effect for the group (*F*_(1, 192)_ = 26.55; *p* < 0.0001). Post hoc pairwise comparisons with Bonferroni’s correction revealed a significant (***p < 0.001; **p < 0.01) difference between the groups (nine mice/group).

Western blot analysis revealed that in primed mice, PDHK1 expression was significantly increased (*t* = 2.454; df = 8; *p < 0.05; unpaired *t* test; five mice/group) in NGF-treated mice (Fig. 1*E*), along with elevated phosphorylation of PDH on S293 (*t* = 2.311; df = 8; *p < 0.05; unpaired *t* test; five mice/group) and S300 (*t* = 2.372; df = 8; *p < 0.05; unpaired *t* test; five mice/group), suggesting that mitochondrial pyruvate oxidation had not been normalized. Similar changes in PDHK1 (*t* = 2.644; df = 8; *p < 0.05; unpaired *t* test; five mice/group) and phospho-PDH (p-pdh S293: *t* = 2.946; df = 8; *p < 0.05; unpaired *t* test; five mice/group; p-pdh S300: *t* = 2.843; df = 8; *p < 0.05; unpaired *t* test; five mice/group) were observed in DRGs 72 h post-PGE_2_ (chronic phase; Fig. 1*F*). Additionally, PDH expression showed a significant reduction (*t* = 2.668; df = 8; *p < 0.05; unpaired *t* test; five mice/group) in the NGF-primed mice (Fig. 1*F*), consistent with the prolonged allodynia observed in response to the direct disruption of mitochondrial pyruvate oxidation (Haque et al., 2024).

Interestingly, while the mitochondrial stress test showed normal OCR levels on Day 7 (Fig. 1*C*), direct measurement of pyruvate oxidation in DRGs revealed a significant reduction in the NGF-treated group (Fig. 1*G*); two-way RM ANOVA revealed a main effect for the time (*F*_(7, 70)_ = 7.752; *p *< 0.0001) and group (*F*_(1, 10)_ = 10.72; *p *= 0.0084); post hoc pairwise comparisons with Bonferroni’s correction revealed a significant difference (*p < 0.05; ***p < 0.001; six mice/group). These data suggest that while pyruvate oxidation does not recover as the allodynia resolves, another compensatory mechanism restores the OCR during the primed phase.

### Inhibition of PDHK1 reverses allodynia in the chronic phase of the hyperalgesic priming model

**Figure 2.**
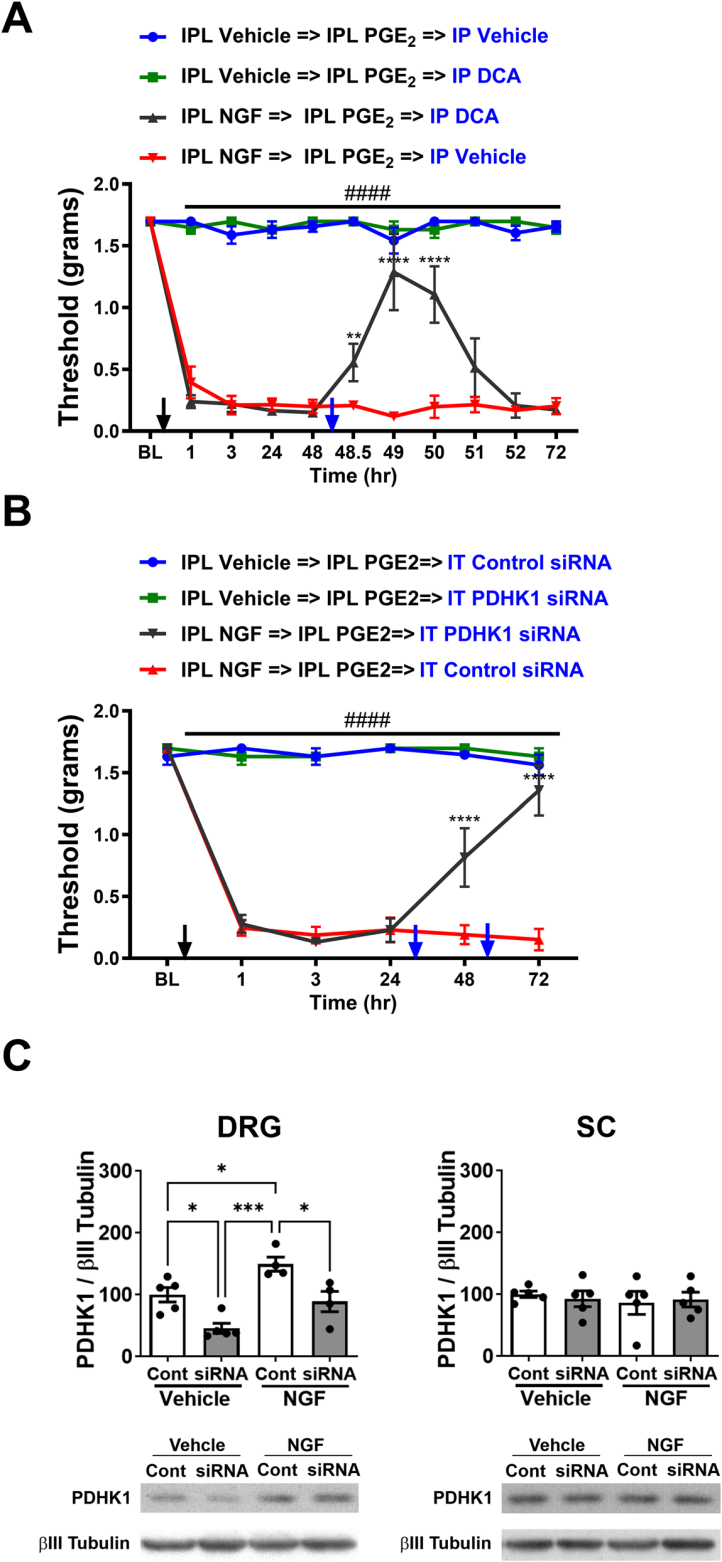
Pharmacological inhibition or siRNA-mediated knockdown of PDHK1 reverses allodynia during the chronic phase of the hyperalgesic priming model. ***A***, Tactile thresholds measured by von Frey filaments in mice treated with IPL vehicle or NGF. Seven days posttreatment (BL, baseline), when allodynia had subsided, IPL PGE_2_ injection precipitated the chronic phase of hyperalgesic priming. Forty-eight hours post-PGE_2_, intraperitoneal administration of the PDHK inhibitor DCA (100 mg/kg) transiently normalized tactile thresholds in NGF-primed mice, while vehicle injection had no effect (IPL NGF→IPL PGE_2_→IP vehicle vs IPL NGF→IPL PGE_2_→IP DCA; *p < 0.05; **p < 0.01; ****p < 0.0001; IPL NGF→IPL PGE_2_→IP vehicle vs IPL vehicle groups; ^####^*p *< 0.0001). ***B***, Tactile thresholds in mice that received IT control or PDHK1-targeting siRNA (1 μg in 5 μl) 24 and 48 h after PGE_2_ treatment. Knockdown of PDHK1 reversed the allodynia in NGF-primed mice (IPL NGF→IPL PGE_2_→IT control siRNA vs IPL NGF→IPL PGE_2_→IT PDHK1 siRNA; ****p < 0.0001; IPL NGF→IPL PGE_2_→IT control siRNA vs IPL vehicle groups; ^####^*p *< 0.0001). ***C***, Confirmation of PDHK1 knockdown using Western blot analysis of PDHK1 expression in L4–L6 DRGs and lumbar SC following IT control or PDHK1 siRNA treatment in vehicle- and NGF-treated mice. PDHK1 siRNA treatment significantly reduced PDHK1 levels in DRGs but not in the SC (*p < 0.05; ***p < 0.001).

We sought to confirm that disruption of mitochondrial pyruvate oxidation drives allodynia in the chronic phase of the hyperalgesic priming model. Forty-eight hours post-PGE_2_ injection, mice primed with either vehicle or NGF were treated with vehicle or DCA, a PDHK inhibitor. NGF-primed mice exhibited profound allodynia, which was reversed by DCA treatment. The effect of DCA peaked at 1 h, and the thresholds were statistically similar to the vehicle-treated group by 4 h (Fig. 2*A*). Two-way RM ANOVA revealed a main effect for the time (*F*_(10, 158)_ = 37.77; *p *< 0.0001) and group (*F*_(3, 16)_ = 345.5; *p *< 0.0001). Post hoc pairwise comparisons with Bonferroni’s correction revealed a significant (^####^*p *< 0.0001) difference between the IPL NGF→IPL PGE_2_→IP vehicle and the IPL vehicle→IPL PGE_2_ group treated with either IP vehicle or IP DCA. Post hoc pairwise comparisons with Bonferroni’s correction also revealed a significant (****p < 0.0001; **p < 0.01; *p < 0.05) difference between the IPL NGF→IPL PGE_2_→IP vehicle and IPL NGF→IPL PGE_2_→IP DCA group (five mice/group).

To further investigate the role of PDHK1, we used siRNA-mediated knockdown. Twenty-four hours post-PGE_2_ treatment, vehicle- and NGF-primed mice received either IT siRNA targeting PDHK1 or control siRNA for 2 consecutive days. PDHK1 siRNA resolved the tactile allodynia in the chronic phase and caused a significant reduction in allodynia relative to the control siRNA-treated group. Knockdown of PDHK1 did not alter the thresholds in the vehicle-primed mice (Fig. 2*B*). Two-way RM ANOVA revealed a main effect for the time (*F*_(5, 79)_ = 59.57; *p *< 0.0001) and group (*F*_(3, 16)_ = 185.7; *p *< 0.0001). Post hoc pairwise comparisons with Bonferroni’s correction revealed a significant (^####^*p *< 0.0001) difference between the IPL NGF→IPL PGE_2_→IT control siRNA and the IPL vehicle→IPL PGE_2_ group injected with either IT control siRNA or IT PDHK1 siRNA. Post hoc pairwise comparisons with Bonferroni’s correction also revealed a significant (****p < 0.0001) difference between the IPL NGF→IPL PGE_2_→IT control siRNA and IPL NGF→IPL PGE_2_→IT PDHK1 siRNA group (five mice/group).

To verify the knockdown of PDHK1, we dissected L4–L6 DRGs and lumbar spinal cords (SCs) 72 h post-PGE_2_ treatment. PDHK1 siRNA effectively reduced the expression of PDHK1 in DRGs; one-way ANOVA revealed a main effect for the group (*F*_(3, 14)_ = 12.73; *p *= 0.003); post hoc pairwise comparisons with Tukey’s correction revealed a significant difference (*p < 0.05; ***p < 0.001; five mice/group) without affecting the section of the SC innervated by the L4–L6 DRGs (Fig. 2*C*). These results demonstrate that disruption of pyruvate oxidation drives the chronic phase of the hyperalgesic priming model, as both pharmacological inhibition and siRNA-mediated knockdown of PDHK1 effectively reversed the allodynia observed in NGF-primed mice.

### LDHA-mediated lactate extrusion drives allodynia in the chronic phase of the hyperalgesic priming model

Pyruvate that is not oxidized in the mitochondria can be converted to lactate via LDHA, which is then extruded from the cell via a symporter that carries a proton, increasing the ECAR (Bender, 2012; Lehninger et al., 2013; Divakaruni et al., 2014; Yang et al., 2014). Since the chronic phase is associated with disruption of mitochondrial pyruvate oxidation, we sought to determine how this impacts ECAR using the glycolysis stress test.

**Figure 3.**
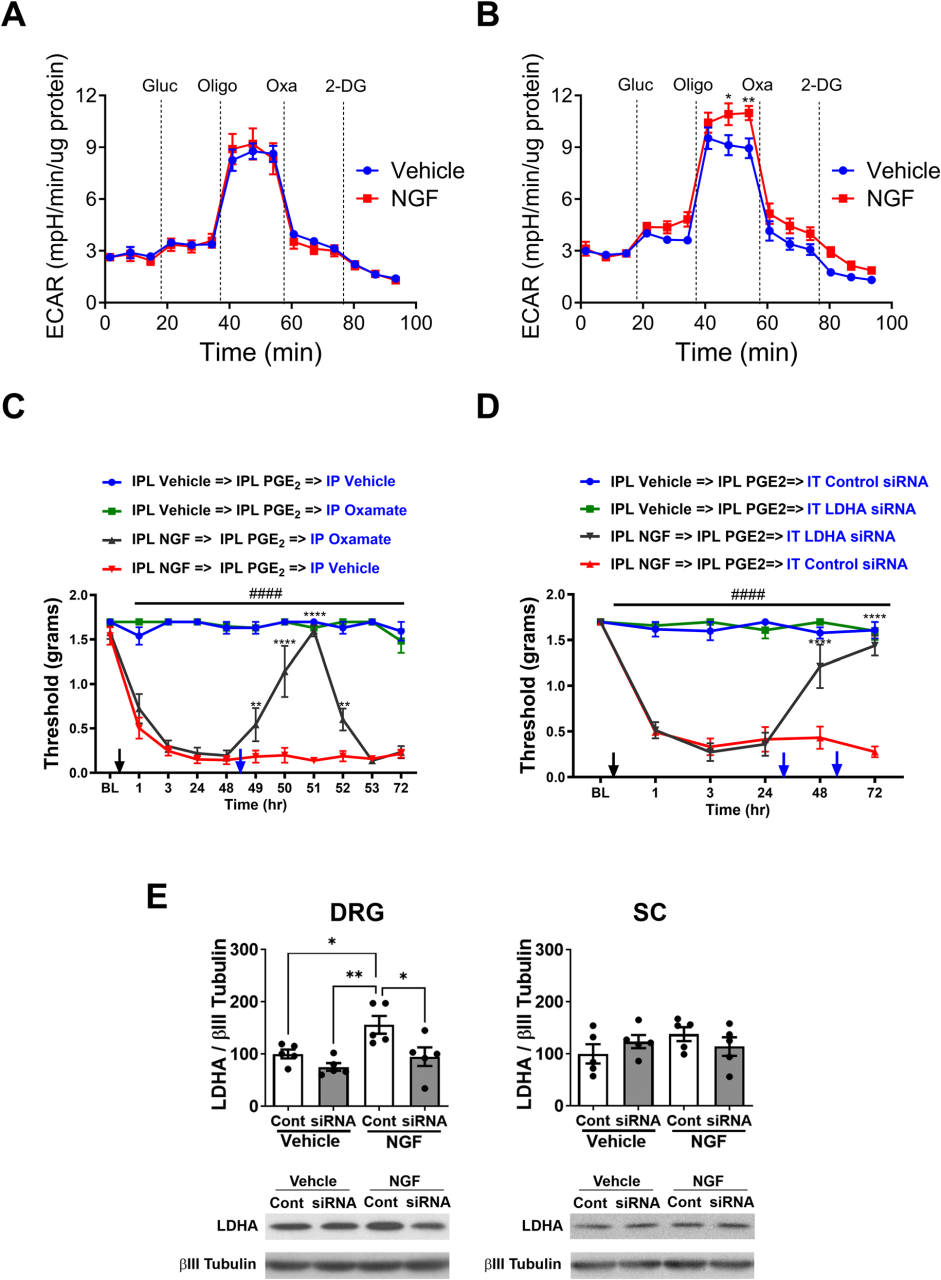
Effect of NGF on glycolysis in lumbar DRGs, and inhibition of LDHA reverses allodynia during the chronic phase of the hyperalgesic priming model. ***A***, ***B***, Glycolysis stress test measured by Seahorse analyzer in lumbar DRGs from NGF- or vehicle-treated mice dissected on Day 7 (***A***) or 3 d post-PGE_2_ injection (***B***). NGF priming increases glycolytic capacity and glycolytic reserve in DRGs in chronic phase (*p < 0.05; **p < 0.01). ***C***, Tactile thresholds measured by von Frey filaments in NGF-primed mice treated with IP vehicle or the glycolysis inhibitor oxamate (500 mg/kg) 48 h after PGE_2_ injection. Oxamate administration reversed the allodynia in NGF-treated mice (IPL NGF→IPL PGE_2_→IP vehicle vs IPL NGF→IPL PGE_2_→IP oxamate; **p < 0.01; ****p < 0.0001; IPL NGF→IPL PGE_2_→IP vehicle vs IPL vehicle groups; ^####^*p *< 0.0001). ***D***, Tactile thresholds in NGF-treated mice that received IT control or LDHA-targeting siRNA (1 μg in 5 μl) 24 and 48 h after PGE_2_ treatment. Knockdown of LDHA reversed the allodynia in NGF-primed mice (IPL NGF→IPL PGE_2_→IT control siRNA vs IPL NGF→IPL PGE_2_→IT LDHA siRNA; ****p < 0.0001; IPL NGF→IPL PGE_2_→IT control siRNA vs IPL vehicle groups; ^####^*p *< 0.0001). ***E***, Confirmation of LDHA knockdown using Western blot analysis of LDHA expression in L4–L6 DRGs and lumbar SC following IT control or LDHA siRNA treatment in vehicle- and NGF-treated mice. LDHA siRNA treatment significantly reduced LDHA levels in DRGs but not in the SC (*p < 0.05; **p < 0.01).

DRGs dissected 7 d postvehicle or NGF treatment showed that the two groups were indistinguishable from each other (Fig. 3*A*) despite the disruption of pyruvate oxidation in primed mice. However, application of oxamate, an LDHA inhibitor, caused a profound reduction of ECAR in both groups, demonstrating that ECAR is driven by LDHA (Fig. 3*A*). This suggests that the compensatory mechanism that restores OCR also limits lactate production or extrusion.

The glycolysis stress test on DRGs dissected from mice 72 h post-PGE_2_ injection revealed that mice primed with NGF showed a significantly higher ECAR in response to oligomycin relative to the vehicle-primed mice. Moreover, application of oxamate profoundly reduced the ECAR in both groups (Fig. 3*B*). Two-way RM ANOVA revealed a main effect for the time (*F*_(14, 140)_ = 241.5; *p* < 0.0001) and group (*F*_(1, 10)_ = 5.048; *p *= 0.0484). Post hoc pairwise comparisons with Bonferroni’s correction revealed a significant (*p < 0.01; **p < 0.05) difference in glycolytic capacity post oligomycin addition (six mice/group).

To determine whether lactate extrusion contributes to allodynia during the chronic phase, we treated vehicle- and NGF-primed mice with either vehicle or oxamate 48 h after PGE_2_ injection. Oxamate alleviated the allodynia in NGF-primed mice, with a peak effect at 3 h that disappeared by 5 h (Fig. 3*C*). Two-way RM ANOVA revealed a main effect for the time (*F*_(10, 160)_ = 32.03; *p *< 0.0001) and group (*F*_(3, 16)_ = 333.5; *p* < 0.0001). Post hoc pairwise comparisons with Bonferroni’s correction revealed a significant (^####^*p *< 0.0001) difference between the IPL NGF→IPL PGE_2_→IP vehicle and the IPL vehicle→IPL PGE_2_ group treated with either IP vehicle or IP oxamate. Post hoc pairwise comparisons with Bonferroni’s correction also revealed a significant (****p < 0.0001; **p < 0.01) difference between the IPL NGF→IPL PGE_2_→IP vehicle and IPL NGF→IPL PGE_2_→IP oxamate group (five mice/group).

Using siRNA, we sought to confirm that LDHA drives the chronic phase. Control or LDHA siRNA was injected intrathecally for 2 consecutive days 24 h post-PGE_2_. Knockdown of LDHA alleviated the allodynia in NGF-primed mice without affecting the vehicle-primed mice (Fig. 3*D*). Two-way RM ANOVA revealed a main effect for the time (*F*_(5, 80)_ = 38.13; *p *< 0.0001) and group (*F*_(3, 16)_ = 135.3; *p *< 0.0001). Post hoc pairwise comparisons with Bonferroni’s correction revealed a significant (^####^*p *< 0.0001) difference between the IPL NGF→IPL PGE_2_→IT control siRNA and the IPL vehicle→IPL PGE_2_ group injected with either IT control siRNA or IT LDHA siRNA. Post hoc pairwise comparisons with Bonferroni’s correction also revealed a significant (****p < 0.0001) difference between the IPL NGF→IPL PGE_2_→IT control siRNA and IPL NGF→IPL PGE_2_→IT LDHA siRNA group (five mice/group). Knockdown in L4–L6 DRGs was confirmed by dissecting DRGs and SC at 72 h and analyzing by Western blots. LDHA siRNA effectively reduced the expression of LDHA in DRGs (Fig. 3*E*); one-way ANOVA revealed a main effect for the group (*F*_(3, 16)_ = 6.533; *p *= 0.0043); post hoc pairwise comparisons with Tukey’s correction revealed a significant difference (*p < 0.05; **p < 0.01; five mice/group). LDHA siRNA normalized the expression of LDHA in NGF-primed mice, which normalized the thresholds. LDHA levels in the SC section innervated by L4–L6 DRGs were not affected (Fig. 3*E*). These results suggest that LDHA-mediated lactate extrusion plays a crucial role in driving allodynia during the chronic phase of the hyperalgesic priming model.

### Pain resolution is associated with increased glutamine oxidation and ASCT2 expression in DRGs, which is lost during the chronic phase of hyperalgesic priming

**Figure 4.**
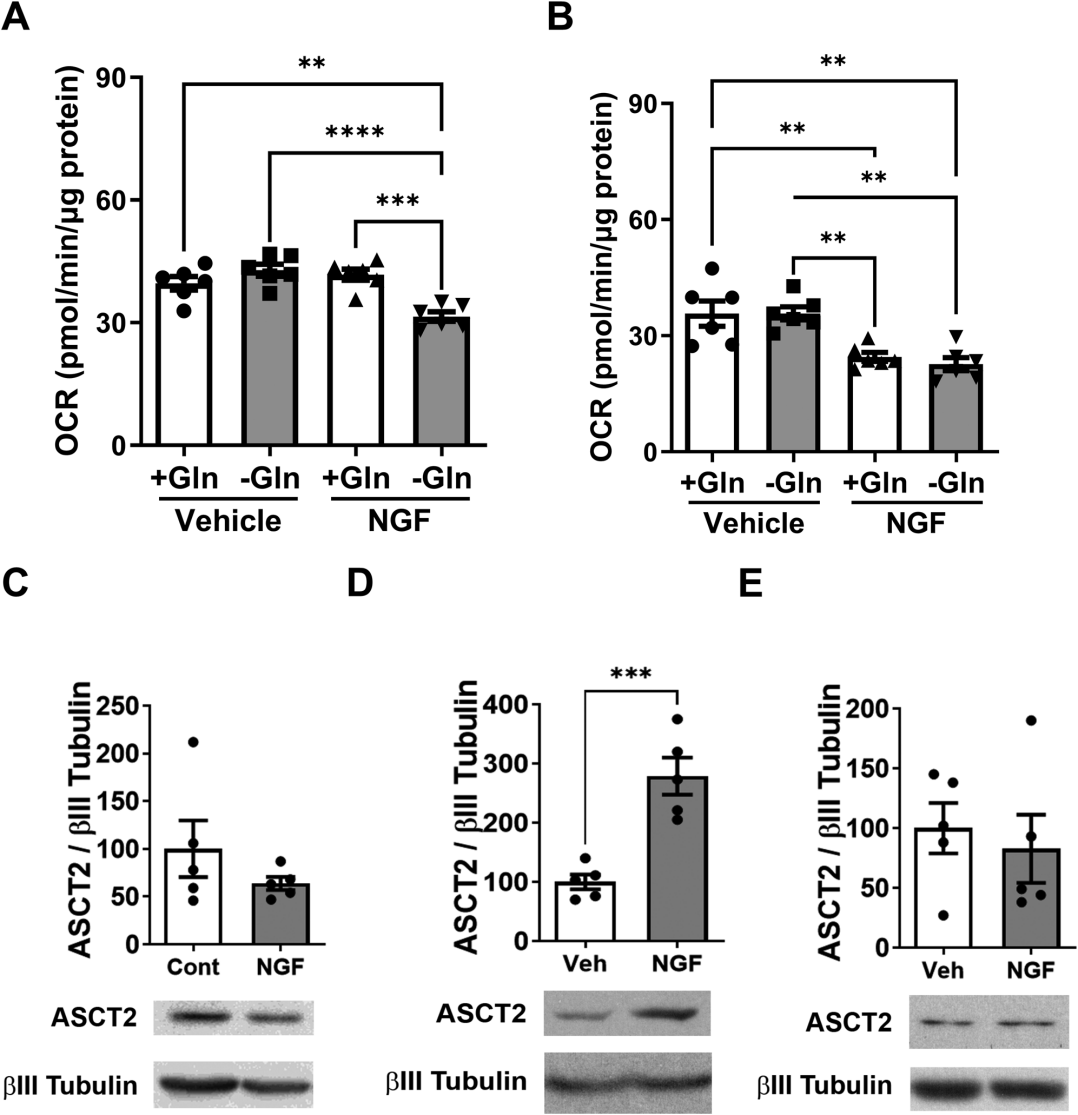
Pain resolution is associated with increased glutamine utilization and ASCT2 expression in lumbar DRGs. ***A***, ***B***, Spare respiratory capacity measured by Seahorse analyzer in lumbar DRGs from NGF- or vehicle-treated mice dissected on Day 7 (***A***) or 3 d post-PGE_2_ injection (***B***). DRGs were incubated in medium with or without glutamine (Gln) during the assay. NGF injection increases spare respiratory capacity in a glutamine-dependent manner in the primed condition which is absent post-PGE_2_ injection (**p < 0.01; ***p < 0.001; ****p < 0.0001). ***C–E***, Western blot analysis of ASCT2 expression in lumbar DRGs from NGF- or vehicle-treated mice on Day 1 (***C***), Day 7 (***D***), or 3 d post-PGE_2_ injection (***E***). NGF injection increases ASCT2 protein levels in DRGs on Day 7 which disappears post-PGE_2_ treatment (***p < 0.001).

To investigate the potential compensatory mechanisms employed by DRGs to maintain normal OCR levels when pyruvate oxidation is disrupted, we conducted mitochondrial stress tests in the presence and absence of glutamine. This allowed us to determine if DRGs become dependent on glutamine oxidation during pain resolution on Day 7. Measurement of spare respiratory capacity revealed that in the presence of glutamine, DRGs from NGF-primed mice showed normal OCR levels. However, in the absence of glutamine, these DRGs exhibited reduced capacity (Fig. 4*A*). One-way ANOVA revealed a main effect for the group (*F*_(3, 20)_ = 12.99; *p *< 0.0001). Post hoc pairwise comparisons with Dunnett’s correction revealed a significant difference (**p < 0.01; ***p < 0.001; ****p < 0.0001; six mice/group). This suggests that DRGs from NGF-primed mice rely on glutamine oxidation to maintain normal OCR levels during pain resolution.

To further explore this phenomenon, we conducted the same experiment on DRGs 72 h post-PGE_2_ injection. Interestingly, we found that DRGs from NGF-primed mice lost the capacity to oxidize glutamine at levels that would compensate for the loss in capacity (Fig. 4*B*). One-way ANOVA revealed a main effect for the group (*F*_(3, 20)_ = 11.18; *p *= 0.0002). Post hoc pairwise comparisons with Tukey’s correction revealed a significant (**p < 0.01; six mice/group). This indicates that the compensatory mechanism of glutamine oxidation is no longer effective during the chronic phase of the hyperalgesic priming model.

Western blot analysis of L4–L6 DRGs 24 h postvehicle or NGF treatment did not show any difference in the expression of ASCT2 (Fig. 4*C*), one of the major glutamine transporters. Seven days postvehicle or NGF injection revealed a significant upregulation of ASCT2 (Fig. 4*D*; *t* = 5.275; df = 8; ***p < 0.001; unpaired *t* test; five mice/group). This upregulation suggests an increased capacity for glutamine uptake in DRGs during pain resolution. However, this increase in ASCT2 expression was absent in DRGs dissected 48 h post-PGE_2_ injection (Fig. 4*E*), indicating a potential loss of the compensatory mechanism during the chronic phase.

These results demonstrate that during pain resolution, DRGs adapt to disruptions in pyruvate oxidation by switching to glutamine oxidation as an alternate fuel source. This compensatory mechanism is associated with increased expression of the glutamine transporter ASCT2. However, this adaptive response appears to be lost during the chronic phase of the hyperalgesic priming model, as evidenced by the reduced capacity for glutamine oxidation and the absence of ASCT2 upregulation post-PGE_2_ injection.

### Knockdown of ASCT2 in primed mice precipitates the chronic phase of hyperalgesic priming, revealing its role in resolution of allodynia and the transition to chronic pain

**Figure 5.**
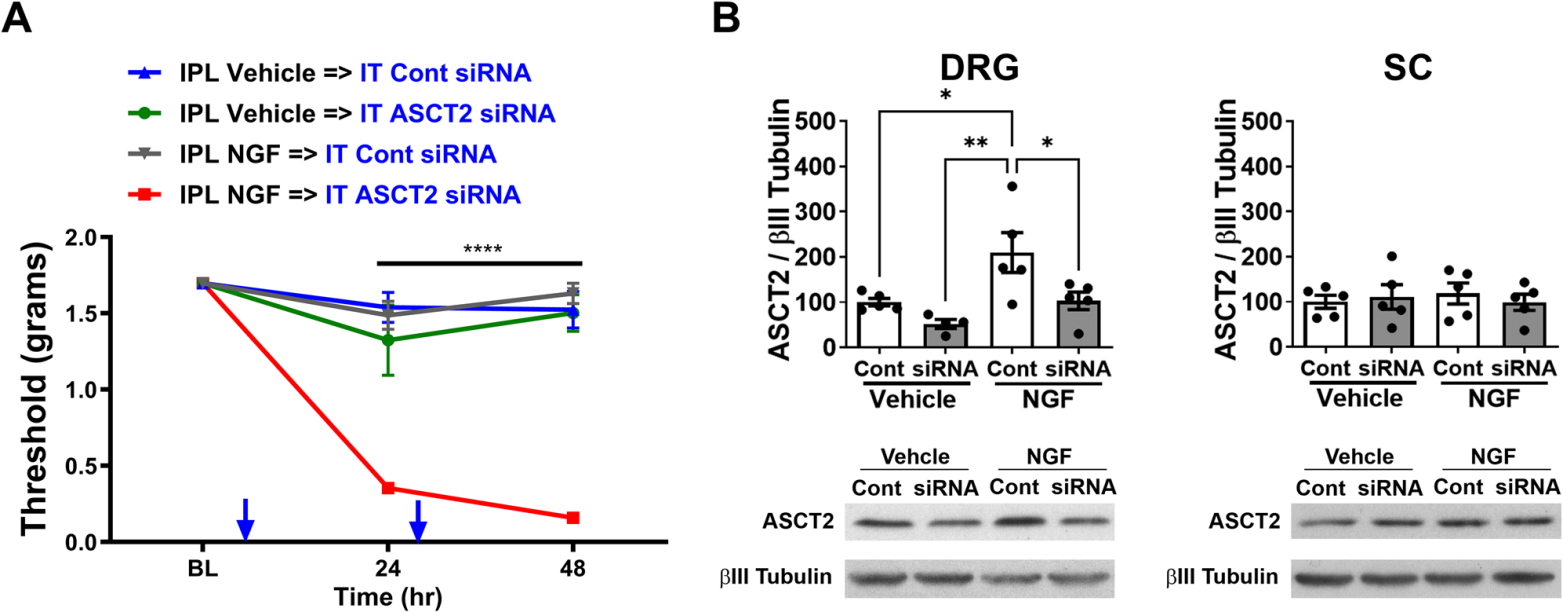
siRNA-mediated knockdown of ASCT2 initiates the chronic phase in primed mice. ***A***, Tactile thresholds measured by von Frey filaments in mice treated with IPL vehicle or NGF. Seven days posttreatment (BL, baseline), when allodynia had subsided, mice received IT control or ASCT2-targeting siRNA (1 μg in 5 μl). Knockdown of ASCT2 precipitated the chronic phase of hyperalgesic priming in NGF-primed mice, while control siRNA had no effect (IPL NGF→IT Cont siRNA vs all other groups; ****p < 0.0001). ***B***, Confirmation of ASCT2 knockdown using Western blot analysis of ASCT2 expression in L4–L6 DRGs and lumbar SC following IT control or ASCT2 siRNA treatment in vehicle- and NGF-treated mice. ASCT2 siRNA treatment significantly reduced ASCT2 levels in DRGs but not in the SC of NGF-primed mice (*p < 0.05; **p < 0.01).

To validate the role of ASCT2 in the resolution of NGF-mediated allodynia and its loss in the transition from acute pain to chronic, we knocked down ASCT2 expression on Day 7 in mice treated with vehicle or NGF. Baseline measurements on Day 7 (BL; Fig. 5*A*) showed that mice did not exhibit any allodynia 7 d postvehicle or NGF treatment. However, intrathecal treatment with ASCT2 siRNA for 2 consecutive days precipitated the chronic phase of the hyperalgesic priming model, confirming the role of ASCT2 in the transition of acute NGF-mediated allodynia to chronic and its involvement in resolving allodynia in the acute phase. Two-way RM ANOVA revealed a main effect for the time (*F*_(2, 46)_ = 36.71; *p *< 0.0001) and group (*F*_(3, 46)_ = 50.39; *p *< 0.0001). Post hoc pairwise comparisons with Bonferroni’s correction revealed a significant (****p < 0.0001) difference between the IPL NGF→IT ASCT2 siRNA with control siRNA and the IPL vehicle group injected with either IT control siRNA or IT ASCT2 siRNA (five mice/group). Notably, knockdown of ASCT2 in vehicle-primed mice did not produce allodynia (Fig. 5*A*).

At the end of the experiment (48 h post-IT siRNA), DRGs and SC were dissected to validate the knockdown of ASCT2. Western blot analysis revealed a significant reduction in ASCT2 expression in the DRGs of mice treated with ASCT2 siRNA compared with control siRNA. In vehicle-treated mice, ASCT2 levels were reduced below control levels without producing allodynia. However, in NGF-primed mice, the normalization of ASCT2 levels to control levels resulted in the development of allodynia (Fig. 5*B*); one-way ANOVA revealed a main effect for the group (*F*_(3, 15)_ = 6.364; *p *= 0.0054); post hoc pairwise comparisons with Tukey’s correction revealed a significant (*p < 0.05; **p < 0.01; five mice/group) difference. This suggests that the allodynia observed in NGF-primed mice following ASCT2 knockdown is driven by the disruption of pyruvate oxidation without the compensatory benefit of enhanced glutamine oxidation. Importantly, ASCT2 expression in the lumbar SC (Fig. 5*B*) was not affected by the siRNA treatment, indicating that the observed effects were specific to the DRGs.

These results demonstrate that ASCT2 plays a crucial role in the resolution of NGF-mediated allodynia, and its loss contributes to the transition from acute to chronic pain. Knockdown of ASCT2 in primed mice precipitates the chronic phase of the hyperalgesic priming model, indicating that ASCT2-mediated glutamine uptake and oxidation are essential for the resolution of acute pain and the prevention of the development of chronic allodynia.

### Glutamine catabolite attenuates glycolytic flux and alleviates allodynia in both acute and chronic phases of the hyperalgesic priming model

To confirm that the catabolism of glutamine is important for the resolution of allodynia, we utilized DKG, a cell-permeable form of alpha-ketoglutarate (Weinberg et al., 2010), which is the end metabolite of glutamine. Glutamine oxidation is a multistep process that involves the transport of glutamine into the cell via ASCT2 (van Geldermalsen et al., 2015), followed by its conversion to alpha-ketoglutarate through two distinct enzymatic reactions catalyzed by glutaminase 1 (GLS1) and glutamate dehydrogenase (Bender, 2012). By employing DKG, we were able to bypass these biochemical steps and directly assess the impact of alpha-ketoglutarate on NGF-mediated allodynia.

**Figure 6.**
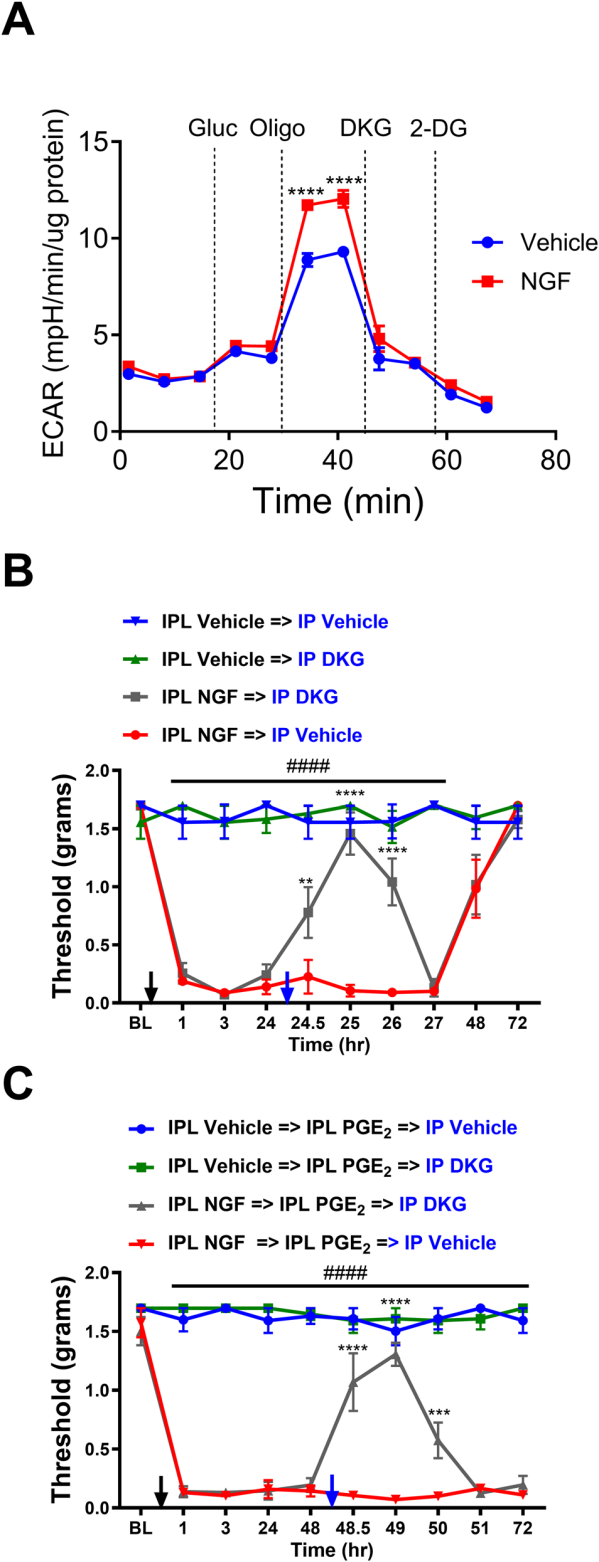
DKG attenuates glycolytic flux and alleviates allodynia in both acute and chronic phases of the hyperalgesic priming model. ***A***, ECAR measured by Seahorse analyzer in lumbar DRGs from NGF- or vehicle-treated mice. NGF significantly increases glycolytic flux compared with vehicle, and this effect is profoundly attenuated by DKG treatment (****p < 0.0001). ***B***, Tactile thresholds measured in mice treated with IPL vehicle or NGF. Intraperitoneal administration of DKG (300 mg/kg) alleviated allodynia in the acute phase post-NGF injection compared with vehicle treatment (IPL NGF→IP DKG vs IPL NGF→IP vehicle; **p < 0.01; ****p < 0.0001; IPL NGF→IP vehicle vs IPL vehicle groups; ^####^*p *< 0.0001; ^###^*p *< 0.001; ^##^*p *< 0.01). ***C***, Tactile thresholds in NGF-primed mice that received IPL PGE_2_ injection to precipitate the chronic phase. IP administration of DKG 48 h post-PGE_2_ alleviated allodynia in the chronic phase compared with vehicle treatment (IPL NGF→IP DKG vs IPL NGF→IP vehicle; ***p < 0.001; ****p < 0.0001; IPL NGF→IP vehicle vs IPL vehicle groups; ^####^*p *< 0.0001).

Glycolysis stress tests on DRGs dissected 48 h post-PGE_2_ showed that NGF treatment caused a significant increase in glycolytic capacity. However, the application of DKG led to a profound reduction in ECAR (Fig. 6*A*). Two-way RM ANOVA revealed a main effect for the time (*F*_(10, 155)_ = 296.2; *p *< 0.0001) and group (*F*_(1, 16)_ = 16.78; *p *= 0.0008). Post hoc pairwise comparisons with Bonferroni’s correction revealed a significant (****p < 0.0001) difference in glycolytic capacity post oligomycin addition (nine mice/group). This reduction is likely due to the interaction of alpha-ketoglutarate with the active site of LDHA. Structural modeling of the LDHA active site has revealed that alpha-ketoglutarate can favorably bind in a spatial configuration similar to that of pyruvate, the canonical substrate of LDHA. This finding suggests that alpha-ketoglutarate and its cell-permeable derivatives, such as DKG, can compete with pyruvate for the LDHA active site (Intlekofer et al., 2015). Consequently, the presence of alpha-ketoglutarate or DKG in the cellular environment may inhibit the conversion of pyruvate to lactate by LDHA, as the enzyme's active site would be occupied by the competing substrate. This competition between alpha-ketoglutarate and pyruvate for the LDHA active site provides a mechanistic explanation for the observed reduction in ECAR when cells are treated with DKG.

To confirm that the glutamine metabolite can alleviate NGF-mediated allodynia, we tested the effect of DKG during the acute and chronic phases of hyperalgesic priming. Treatment of mice with DKG (300 mg/kg, i.p.) 24 h postvehicle or NGF injection alleviated NGF-induced allodynia for several hours (Fig. 6*B*). Two-way RM ANOVA revealed a main effect for the time (*F*_(9, 142)_ = 26.64; *p *< 0.0001) and group (*F*_(3, 16)_ = 221.1; *p *< 0.0001). Post hoc pairwise comparisons with Bonferroni’s correction revealed a significant (^####^*p *< 0.0001; ^###^*p *< 0.001; ^##^*p *< 0.01) difference between the IPL NGF→IP vehicle and the IPL vehicle group treated with either IP vehicle or IP DKG. Post hoc pairwise comparisons with Bonferroni’s correction also revealed a significant (****p < 0.0001; **p < 0.01) difference between the IPL NGF→IP vehicle and IPL NGF→IP DKG group (five mice/group). Moreover, treatment with DKG 48 h post-PGE_2_ alleviated allodynia in NGF-primed mice (Fig. 6*C*). Two-way RM ANOVA revealed a main effect for the time (*F*_(9, 144)_ = 31.33; *p *< 0.0001) and group (*F*_(3, 16)_ = 652.2; *p* < 0.0001). Post hoc pairwise comparisons with Bonferroni’s correction revealed a significant (^####^*p *< 0.0001) difference between the IPL NGF→IPL PGE_2_→IP vehicle and the IPL vehicle→IPL PGE_2_ group injected with either IP vehicle or IP DKG. Post hoc pairwise comparisons with Bonferroni’s correction also revealed a significant (****p < 0.0001; ***p < 0.001) difference between the IPL NGF→IPL PGE_2_→IP vehicle and IPL NGF→IPL PGE_2_→IP DKG (five mice/group).

These data demonstrate that DKG, a cell-permeable form of the glutamine catabolite, effectively attenuates glycolytic flux and alleviated allodynia in both the acute and chronic phases of the hyperalgesic priming model.

### Targeting metabolic pathways alleviates chronic pain in the hyperalgesic priming model

The CPA assay was employed to investigate the affective and motivational aspects of pain in the chronic phase of the hyperalgesic priming model. This assay is particularly useful as it measures an animal's aversion to an environment associated with a painful or aversive stimulus (Navratilova and Porreca, 2014). In this study, we used glucose administration as the aversive stimulus, based on several lines of evidence suggesting a link between glucose metabolism and chronic pain. First, our previous work has shown that the chemotherapeutic agent bortezomib induces aerobic glycolysis in DRG neurons, leading to increased glycolytic flux. Furthermore, we demonstrated that glucose administration produces CPA in bortezomib-treated mice, indicating that increased glucose metabolism can contribute to the affective and motivational aspects of chemotherapy-induced neuropathic pain (Ludman and Melemedjian, 2019b). Additionally, a clinical report has documented higher plasma glucose values during spontaneous migraine attacks, suggesting the potential role of glucose metabolism in chronic pain (D. G. Zhang et al., 2020). Together, these findings led us to hypothesize that promoting glycolytic flux through glucose administration would exacerbate NGF-induced pain and that this effect could be measured using the CPA assay.

**Figure 7.**
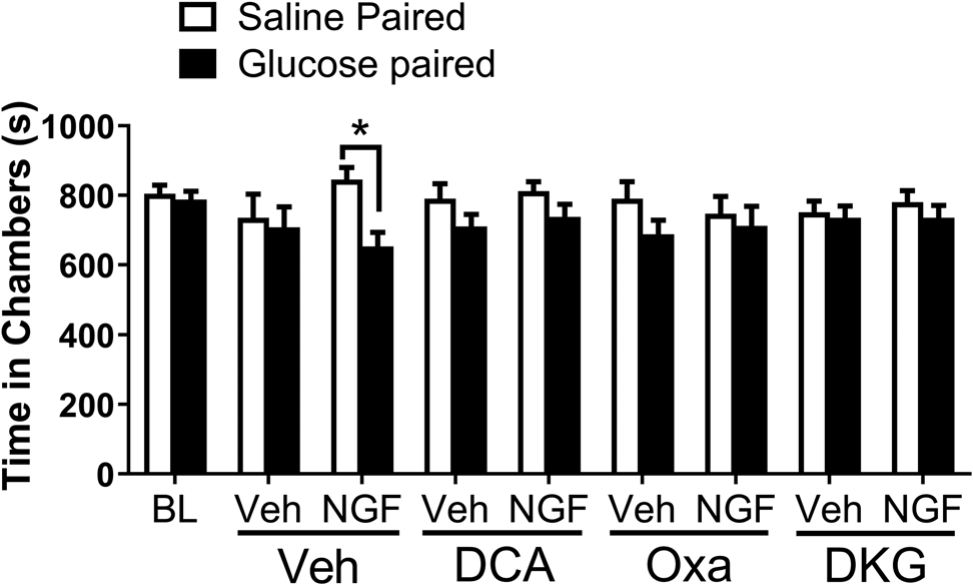
DCA, oxamate, and DKG block glucose-induced CPA in the chronic phase of the hyperalgesic priming model. CPA was assessed using a two-chamber paradigm, where one chamber was paired with saline and the other with glucose (2 g/kg, i.p.) in vehicle or NGF-injected mice during the chronic phase. Glucose induces a significant place aversion compared with saline in NGF-primed mice (glucose paired vs saline paired; *p < 0.05). Treatment with DCA (100 mg/kg), oxamate (Oxa, 500 mg/kg), or DKG (300 mg/kg) prior to glucose pairing blocked the glucose-induced CPA in NGF-primed mice.

Mice were subjected to a two-chamber paradigm, where one chamber was paired with saline and the other with glucose (2 g/kg, i.p.) during the chronic phase of the hyperalgesic priming model. NGF-primed mice showed a significant aversion to the glucose-paired chamber compared with the saline-paired chamber (Fig. 7), indicating that glucose administration induced an aversive state in these mice. Two-way RM ANOVA revealed a main effect for the group (*F*_(1, 182)_ = 11.50; *p *= 0.0009). Post hoc pairwise comparisons with Bonferroni’s correction revealed a significant (*p < 0.05) difference between the NGF and vehicle group. In contrast, vehicle-primed mice did not exhibit aversion to either chamber, suggesting that glucose alone did not influence their behavior.

To assess the efficacy of potential analgesic compounds, we administered DCA (100 mg/kg), oxamate (500 mg/kg), and DKG (300 mg/kg) 1, 2, and 1 h prior to glucose pairing, respectively, to coincide with their peak antiallodynic effects as determined in previous experiments (Figs. 2, 3, 6). Remarkably, treatment with DCA, oxamate, or DKG effectively blocked the glucose-induced CPA in NGF-primed mice (Fig. 7).

The ability of DCA to block glucose-induced CPA validates the notion that mitochondrial pyruvate oxidation is disrupted in the chronic phase of the hyperalgesic priming model. This disruption can lead to the conversion of glucose-derived pyruvate to lactate, which is extruded from the cells along with a proton. The extracellular accumulation of lactate and protons can activate a multitude of pronociceptive receptors and channels on DRG neurons, causing pain. The efficacy of oxamate, a lactate dehydrogenase inhibitor, in blocking glucose-induced CPA further supports this mechanism by demonstrating that the prevention of lactate generation can alleviate pain. Finally, the ability of DKG, a glutamine catabolite, to block glucose-induced CPA suggests that enhancing glutamine metabolism can compensate for the disrupted pyruvate oxidation and reduce lactate production, ultimately leading to pain relief. Together, these results confirm that disrupted pyruvate oxidation plays a critical role in the maintenance of the chronic phase, and the alleviation of pain by a glutamine catabolite underscores the potential importance of enhanced glutamine metabolism in the resolution of chronic pain states.

### Glutamine catabolite counteracts the pronociceptive effects of disrupted mitochondrial pyruvate oxidation in sensory neurons

We have previously demonstrated that disruption of mitochondrial pyruvate oxidation is sufficient to cause prolonged allodynia and enhance calcium responses in DRG neurons, unlike NGF-induced allodynia, which resolves within 72 h (Haque et al., 2024) despite being driven by impaired mitochondrial pyruvate oxidation. Enhanced glutamine oxidation appears to be the mechanism by which NGF-induced allodynia resolves. To investigate whether the glutamine catabolite DKG can attenuate the calcium responses and allodynia caused by the disruption of mitochondrial pyruvate oxidation, we performed calcium imaging experiments in dissociated DRG neurons and assessed mechanical allodynia in a mouse model of PDP1 knockdown-induced chronic nociception.

**Figure 8.**
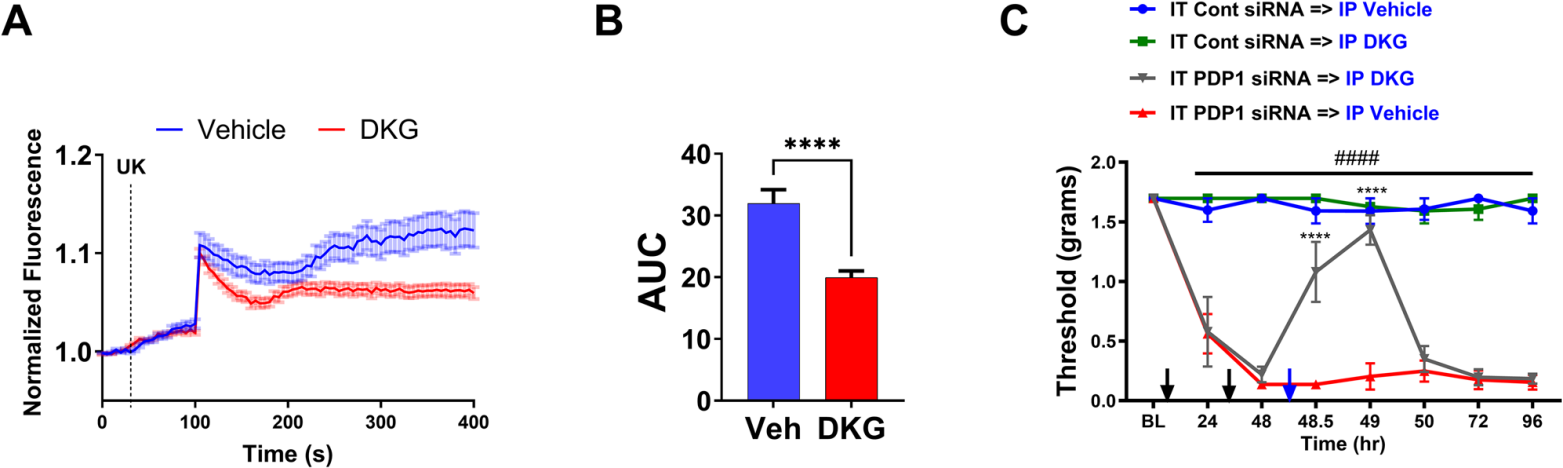
DKG attenuates UK-mediated calcium influx and alleviates PDP1 knockdown-induced allodynia. ***A***, Cumulative average of calcium imaging traces showing the changes in Fluo4 fluorescence in dissociated DRG neurons. Cells were treated with either vehicle or DKG (1 mM), and baseline measurements were performed for 25 s. UK5099 (5 µM) was added to both groups, and the Fluo4 fluorescence was recorded for 400 s. ***B***, AUC analysis of UK5099 revealed that DRG neurons pretreated with DKG display a significantly lower AUC of calcium responses than the vehicle (Veh) pretreated group (****p < 0.0001). ***C***, PDP1 siRNA-induced (black arrows) allodynia was inhibited for several hours by DKG (blue arrow; 300 mg/kg, i.p.; IT PDP1 siRNA→IP DKG vs IT PDP1 siRNA→IP vehicle; ****p < 0.0001; IT PDP1siRNA→IP vehicle vs IT Cont siRNA groups; ^####^*p *< 0.0001).

In calcium imaging experiments, the addition of UK5099 (5 µM, a blocker of the mitochondrial pyruvate importer) at 100 s (Fig. 8*A*) leads to an increase in intracellular calcium. This rise in calcium is due to the blockade of mitochondrial pyruvate import, which shifts metabolism toward increased lactate production. The subsequent extrusion of lactate, along with protons, via the monocarboxylate transporter creates an extracellular acidification that activates various pronociceptive channels and receptors. This mechanism is consistent with our previous findings showing that inhibition of LDHA with oxamate prevents this calcium influx (Haque et al., 2024). However, DRG neurons pretreated with DKG (1 mM) exhibited significantly lower calcium responses to UK5099, compared with vehicle-pretreated neurons (Fig. 8*A*,*B*; AUC, *t* = 5.249; df = 84; ****p < 0.0001; unpaired *t* test; 37–49 neurons/group).

To assess the effect of DKG on allodynia caused by the disruption of mitochondrial pyruvate oxidation in vivo, we injected the mice intrathecally with siRNA targeting PDP1, a key regulator of mitochondrial pyruvate oxidation, for 2 consecutive days. IT PDP1 siRNA treatment can cause profound allodynia lasting months (Haque et al., 2024). Administration of DKG (300 mg/kg, i.p.) 48 h after IT PDP1 siRNA significantly reduced the allodynia, with the effect peaking at 1 h posttreatment (Fig. 8*C*). Two-way RM ANOVA revealed a main effect for the time (*F*_(7, 110)_ = 28.16; *p *< 0.0001) and group (*F*_(3, 16)_ = 253.0; *p *< 0.0001). Post hoc pairwise comparisons with Bonferroni’s correction revealed a significant (^####^*p *< 0.0001) difference between the IT PDP1 siRNA→IP vehicle and the IT control siRNA group injected with either IP vehicle or IP DKG. Post hoc pairwise comparisons with Bonferroni’s correction also revealed a significant (****p < 0.0001) difference between the IT PDP1 siRNA→IP vehicle and IT PDP siRNA→IP DKG (five mice/group).

These results demonstrate that the glutamine catabolite DKG can effectively attenuate the calcium responses and allodynia caused by the disruption of mitochondrial pyruvate oxidation. The ability of DKG to reduce UK5099-induced calcium influx in DRG neurons and alleviate PDP1 knockdown-induced allodynia supports the notion that enhanced glutamine oxidation plays a crucial role in the resolution of pain states by counteracting the pronociceptive effects of impaired mitochondrial pyruvate oxidation in sensory neurons.

### ASCT2 is essential for the resolution of NGF- and incision-induced allodynia

**Figure 9.**
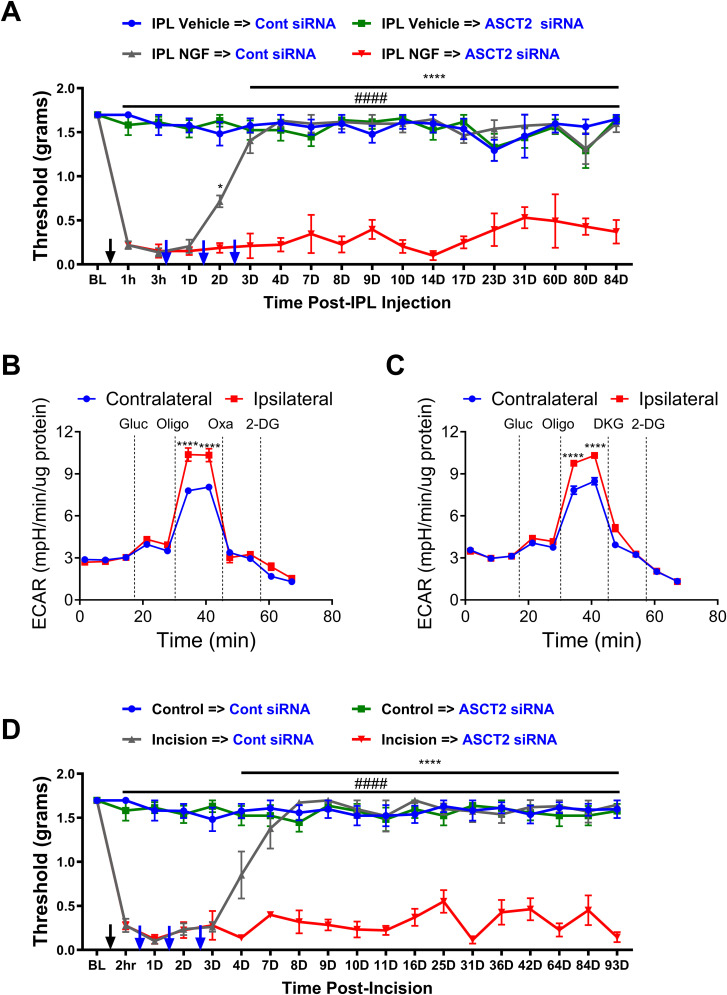
ASCT2 siRNA treatment prevents the resolution of NGF and incision-induced allodynia. ***A***, Intrathecal injection of ASCT2 siRNA at 3 h, Day 1, and Day 3 post-NGF administration prevents the resolution of NGF-induced allodynia for at least 84 d compared with control siRNA (IPL NGF→ASCT2 siRNA vs IPL NGF→Cont siRNA; *p < 0.05; ****p < 0.0001; IPL NGF→ASCT2 siRNA vs IPL vehicle groups; ^####^*p *< 0.0001). ***B***, Plantar incision increases glycolytic flux in ipsilateral lumbar DRGs relative to the contralateral DRGS as measured by glycolysis stress test. Oxamate (Oxa) treatment blocks the incision-induced increase in glycolytic flux (****p < 0.0001). ***C***, DKG treatment also blocks the incision-induced increase in glycolytic flux in lumbar DRGs (****p < 0.0001). ***D***, Intrathecal injection of ASCT2 siRNA at 2 h, Day 1, and Day 2 postincision prevents the resolution of incisional pain for at least 93 d compared with control siRNA (incision→ASCT2 siRNA vs incision→Cont siRNA; ****p < 0.0001; incision→ASCT2 siRNA vs control groups; ^####^*p *< 0.0001).

To determine if ASCT2 knockdown would prevent the resolution of NGF-induced allodynia, mice were injected with either IPL vehicle or NGF and received intrathecal control or ASCT2-targeting siRNA for 3 consecutive days. In mice that received NGF and control siRNA, the allodynia resolved by 72 h, consistent with all previous results. However, silencing of ASCT2 prevented the resolution of NGF-induced allodynia for at least 84 d (Fig. 9*A*). Two-way RM ANOVA revealed a main effect for the time (*F*_(17, 271)_ = 13.34; *p *< 0.0001) and group (*F*_(3, 16)_ = 285.3; *p *< 0.0001). Post hoc pairwise comparisons with Bonferroni’s correction revealed a significant (^####^*p *< 0.0001) difference between the IPL NGF→ASCT2 siRNA and the IPL vehicle group injected with either control siRNA or ASCT2 siRNA. Post hoc pairwise comparisons with Bonferroni’s correction also revealed a significant (****p < 0.0001 and *p < 0.05) difference between the IPL NGF→ASCT2 siRNA and IPL NGF→control siRNA (five mice/group). Notably, ASCT2 siRNA did not produce allodynia in the vehicle-pretreated group.

We have previously shown that plantar incision is associated with an increase in PDHK1 expression in DRGs and that the allodynia is alleviated by DCA (Haque et al., 2024). Hence, we next explored whether plantar incision produces the same glycolytic phenotype as IPL NGF injection. Indeed, plantar incision caused a significant increase in ECAR in DRGs ipsilateral to the incision, as measured by a glycolysis stress test (Fig. 9*B,C*). Moreover, application of oxamate [Fig. 9*B*; two-way RM ANOVA revealed a main effect for the time (*F*_(10, 110)_ = 384.6; *p *< 0.0001) and group (*F*_(1, 10)_ = 12.90; *p *= 0.0049); post hoc pairwise comparisons with Bonferroni’s correction revealed a significant (****p < 0.0001) difference in glycolytic capacity post oligomycin addition (six mice/group)] or DKG [Fig. 9*C*; two-way RM ANOVA revealed a main effect for the time (*F*_(10, 97)_ = 517.3; *p *< 0.0001) and group (*F*_(1, 10)_ = 30.79; *p *= 0.0002); post hoc pairwise comparisons with Bonferroni’s correction revealed a significant (****p < 0.0001) difference in glycolytic capacity post oligomycin addition (six mice/group)] profoundly abrogated the ECAR.

Collectively, these results demonstrate that NGF and plantar incision share many of the biochemical and metabolic changes. Hence, we wanted to test if ASCT2 silencing prevents the resolution of allodynia in the plantar incision model. Indeed, treatment of mice with IT ASCT2 siRNA for 3 consecutive days prevented the resolution of allodynia for at least 93 d (Fig. 9*D*). Two-way RM ANOVA revealed a main effect for the time (*F*_(18, 281)_ = 17.35; *p *< 0.0001) and group (*F*_(3, 16)_ = 298.7; *p *< 0.0001). Post hoc pairwise comparisons with Bonferroni’s correction revealed a significant (^####^*p *< 0.0001) difference between the incision→ASCT2 siRNA and the control group injected with either control siRNA or ASCT2 siRNA. Post hoc pairwise comparisons with Bonferroni’s correction also revealed a significant (****p < 0.0001) difference between the incision→ASCT2 siRNA and incision→control siRNA (five mice/group).

These findings suggest that ASCT2-mediated glutamine transport is crucial for the resolution of allodynia in both NGF- and incision-induced pain models. The inability of sensory neurons to resolve allodynia following ASCT2 knockdown indicates that glutamine metabolism plays a critical role in the endogenous mechanisms that promote the resolution of chronic pain states.

## Discussion

This study significantly advances our understanding of metabolic adaptations in sensory neurons during pain progression, resolution, and chronification. Building upon our previous work, which demonstrated that NGF reprograms sensory neuron metabolism leading to suppressed mitochondrial pyruvate oxidation and pervasive transcriptomic changes (Haque et al., 2024), we now uncover a novel compensatory mechanism involving enhanced glutamine oxidation and upregulation of a major glutamine transporter ASCT2.

A key finding of the current work is that contrary to what might be expected, pyruvate metabolism does not return to normal even after the apparent resolution of allodynia. Instead, we discovered that sensory neurons adapt to this persistent metabolic perturbation through an anaplerotic response, upregulating ASCT2 to enhance glutamine uptake and oxidation. This adaptive mechanism allows neurons to maintain normal oxygen consumption rates and resolve hypersensitivity despite ongoing impairment of pyruvate oxidation.

Importantly, our previous results indicate that this compensatory mechanism is not simply a consequence of disrupted mitochondrial pyruvate oxidation. Direct disruption of pyruvate oxidation leads to persistent pain that does not resolve (Haque et al., 2024). In contrast, NGF and incision-induced pain do resolve, suggesting that these stimuli activate additional pathways that induce ASCT2 expression and mediate pain resolution. This distinction is crucial for understanding the complex interplay between nociceptive signaling and metabolic adaptations in sensory neurons.

The concepts of cataplerosis and anaplerosis are central to understanding these metabolic dynamics (Owen et al., 2002). Cataplerosis, the net efflux of intermediates from the Krebs cycle, is exemplified by the NGF- and plantar incision-induced suppression of pyruvate entry into the cycle (Haque et al., 2024). This cataplerotic effect, driven by increased PDHK1 expression and PDH phosphorylation, not only compromises mitochondrial energy production but also alters the levels of key metabolic intermediates.

The importance of maintaining appropriate levels of Krebs cycle intermediates cannot be overstated. These metabolites are crucial for a multitude of biochemical reactions essential for cellular homeostasis, extending far beyond their role in energy production (DeBerardinis and Thompson, 2012). Acetyl-CoA serves as a critical substrate for various acetylation reactions throughout the cell. It modulates the acetylation status of histones, metabolic enzymes, transcription factors, and structural proteins (Choudhary et al., 2014). These acetylation events play crucial roles in regulating gene expression, protein stability, localization, and interactions.

Alpha-ketoglutarate functions as a cosubstrate for a wide array of dioxygenase enzymes (Hausinger, 2004). These α-ketoglutarate-dependent dioxygenases participate in diverse cellular processes including RNA, DNA, and histone demethylation, fatty acid metabolism, and oxygen sensing (Loenarz and Schofield, 2008). The prolyl hydroxylases that regulate hypoxia-inducible factor stability are also α-ketoglutarate-dependent, linking this metabolite to cellular responses to hypoxia and oxidative stress (Semenza, 2012).

Succinate plays a vital role in heme biosynthesis, impacting numerous heme-containing proteins. These include cytochromes in the electron transport chain, various P450 enzymes involved in drug metabolism, and enzymes like nitric oxide synthase, catalases, and peroxidases. Heme as a prosthetic group is essential for the function of certain proteins that regulate mitochondrial targeting, transcriptional regulation, protein degradation, and miRNA regulation (Furuyama et al., 2007). Succinyl-CoA is also involved in the metabolism of certain amino acids and in the synthesis of succinylated proteins, a posttranslational modification important in cellular function (Z. Zhang et al., 2011).

Oxaloacetate and malate, as key components of the malate–aspartate shuttle, are crucial for maintaining the NAD+/NADH balance between cytosol and mitochondria. This balance affects numerous redox-dependent processes throughout the cell. Additionally, these metabolites play a vital role in amino acid and neurotransmitter metabolism in the nervous system (Lehninger et al., 2013).

Citrate, when exported from the mitochondria, serves as the primary source of acetyl-CoA for fatty acid and cholesterol biosynthesis in the cytosol (Lehninger et al., 2013). It also acts as an allosteric inhibitor of phosphofructokinase, a key regulatory enzyme in glycolysis, thereby playing a role in the coordination of glucose and fatty acid metabolism (Usenik and Legisa, 2010).

Our findings also shed light on the complex interplay between glycolysis, mitochondrial metabolism, and pain signaling. The ability of DKG to compete with pyruvate for LDHA binding (Intlekofer et al., 2015) provides a mechanistic explanation for its efficacy in reducing extracellular acidification and alleviating pain. This competitive inhibition of lactate production represents a novel mechanism by which anaplerotic substrates can modulate nociceptive signaling, beyond their role in supporting mitochondrial metabolism and influencing gene expression.

Depletion of these intermediates due to cataplerosis can have wide-ranging effects on cellular function, including alterations in gene expression, protein function, metabolic flux, and cellular signaling pathways. This might explain why the anaplerotic response we have uncovered, mediated by ASCT2 upregulation and enhanced glutamine oxidation, is so critical. This response replenishes Krebs’ cycle intermediates by increasing the influx of glutamine-derived α-ketoglutarate. This compensatory mechanism likely helps mitigate the broad cellular impacts of cataplerosis, which might include the transcriptomic alterations we previously reported. Our demonstration that the glutamine catabolite α-ketoglutarate attenuates glycolytic flux and alleviates allodynia in both acute and chronic pain phases suggests mechanisms that support anaplerosis are worth further investigation as potential therapeutics.

Our data demonstrate that timely ASCT2 upregulation during pain resolution represents a critical window of opportunity for preventing chronic pain development. Disruption of this anaplerotic response through ASCT2 knockdown prevents the resolution of both NGF- and incision-induced allodynia, leading to persistent pain lasting several months. This finding highlights the essential role of metabolic adaptation in pain resolution and suggests that failure to engage or maintain this compensatory mechanism in a timely manner may lead to pain chronification.

The loss of ASCT2 upregulation during the chronic phase of hyperalgesic priming provides important insights into the metabolic basis of chronic pain. We propose that chronic pain represents a state where ongoing cataplerosis is no longer adequately compensated by anaplerotic processes. This metabolic maladaptation may be the result of persistent inflammatory signaling, epigenetic changes, or other factors that impair the sensory neurons’ ability to maintain enhanced glutamine metabolism in the face of chronic pyruvate oxidation deficits. Crucially, our ASCT2 knockdown experiments in primed mice show that this loss of compensatory glutamine metabolism is not merely associated with chronic allodynia but is causally involved in its development.

The efficacy of metabolic interventions in alleviating both evoked nociceptive behaviors and affective pain responses, as demonstrated in our CPA experiments, underscores the broad impact of metabolic dysfunction on multiple dimensions of the pain experience. The ability of DCA, oxamate, and DKG to block glucose-induced place aversion in NGF-primed mice suggests that targeting both cataplerotic and anaplerotic pathways could provide relief for both sensory and affective components of chronic pain.

The metabolic changes observed in DRG neurons are likely initiated by NGF signaling through its high-affinity receptor TrkA, expressed on a subset of nociceptive neurons. However, the extrusion of metabolites like lactate and protons from these neurons could potentially have paracrine effects on adjacent cells, regardless of their TrkA expression. In the incision model, the release of multiple factors postinjury may activate a broader range of nociceptors beyond those expressing TrkA, potentially leading to more widespread metabolic alterations. Further research using cell-type–specific approaches is needed to elucidate the precise role of TrkA signaling and distinguish between direct NGF effects and secondary responses in both models. It is also important to note that this study was conducted exclusively in male mice. Future studies should investigate these mechanisms in female mice to determine if there are any sex-specific differences in pain resolution and chronification pathways.

**Figure 10.**
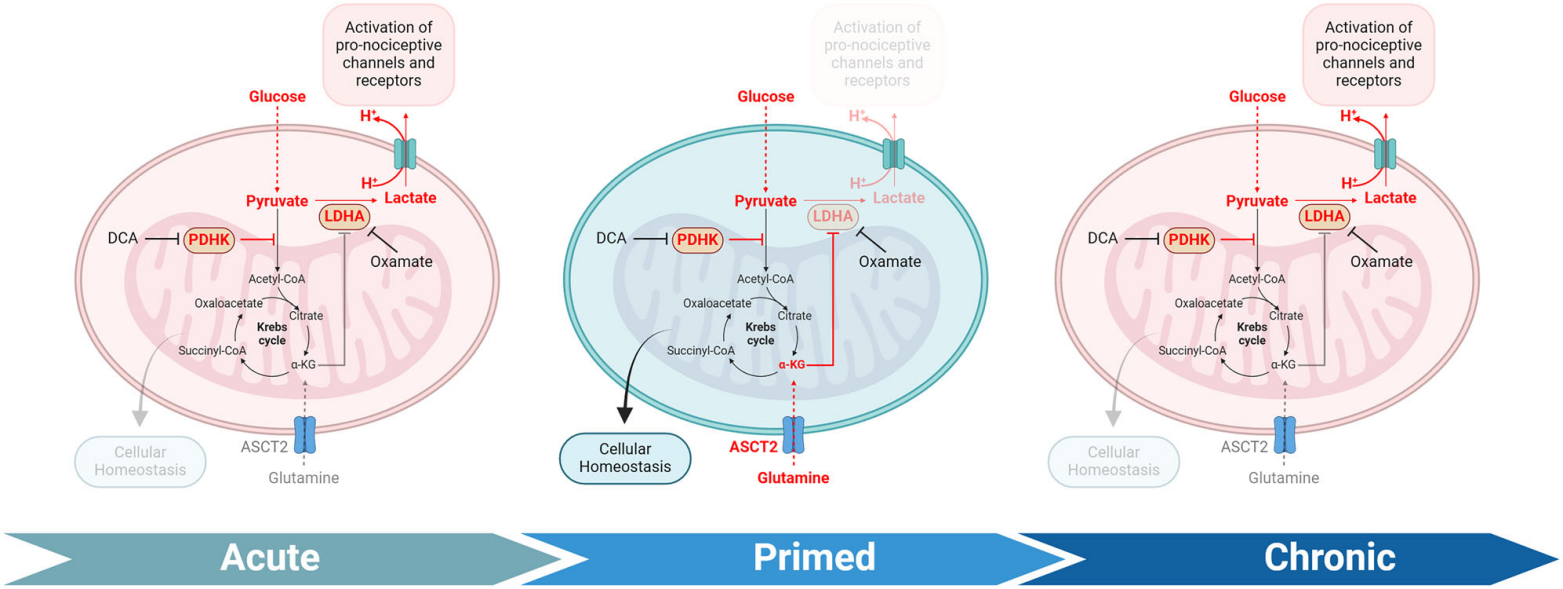
Metabolic adaptations in sensory neurons during pain progression, resolution, and chronification. This schematic illustrates three phases: acute, primed, and chronic. In the acute phase, NGF or plantar incision enhance PDHK expression, limiting mitochondrial pyruvate oxidation and increasing lactate production. Extrusion of lactate and protons can activate pronociceptive channels and receptors. The primed phase shows pain resolution through increased glutamine transporter ASCT2 expression, enabling enhanced glutamine oxidation that maintains cellular homeostasis despite persistent PDHK activity. Moreover, glutamine-derived α-ketoglutarate (α-KG) interferes with lactate production. The chronic phase, triggered by IPL PGE_2_, depicts loss of ASCT2 upregulation, decreased glutamine utilization, and renewed lactate production. Pharmacological interventions include DCA (inhibits PDHK) and oxamate (inhibits LDHA). This model highlights the role of metabolic adaptations, particularly glutamine metabolism, in pain resolution and chronification. Created with BioRender.com.

In conclusion, our study reveals that pain resolution occurs not through normalization of pyruvate metabolism, but through a compensatory upregulation of ASCT2-mediated glutamine metabolism. This adaptive mechanism allows sensory neurons to maintain function and resolve pain sensitivity despite persistent impairment of mitochondrial pyruvate oxidation. Importantly, this compensatory response is not simply a consequence of disrupted pyruvate oxidation but is likely triggered by specific signaling pathways activated by NGF and incision. The eventual failure of this compensatory mechanism may underlie the transition to chronic pain states (Fig. 10). These findings significantly expand our understanding of the metabolic underpinnings of pain progression and chronification, identifying novel therapeutic targets for pain management. Future research should focus on developing strategies to enhance or preserve ASCT2 function and glutamine metabolism in sensory neurons as potential treatments for chronic pain conditions. Additionally, investigating the molecular mechanisms regulating ASCT2 expression and activity in sensory neurons could uncover new approaches for maintaining metabolic adaptability and promoting pain resolution in chronic pain states.

## Data Availability

All data are available upon request.
